# Detection and localization of radiation-induced pneumonitis using $T_{2}$-mapping magnetic resonance imaging

**DOI:** 10.1016/j.phro.2025.100878

**Published:** 2025-11-29

**Authors:** Rabea Klaar, Kaltra Begaj, Moritz Rabe, Stefanie Corradini, Chukwuka Eze, Claus Belka, Bastian Sabel, Guillaume Landry, Christopher Kurz, Julien Dinkel

**Affiliations:** a Department of Radiology, LMU University Hospital, LMU Munich, Munich, 81377, Germany; b Comprehensive Pneumology Center (CPC-M), Member of the German Center for Lung Research (DZL), Munich, 81377, Germany; c Department of Radiation Oncology, LMU University Hospital, LMU Munich, Munich, 81377, Germany; d Now at Department of Radiation Oncology, Universitätsklinikum Erlangen, Friedrich-Alexander-Universität Erlangen-Nürnberg (FAU), Erlangen, 91054, Germany; e German Cancer Consortium (DKTK), partner site Munich, a partnership between DKFZ and LMU University Hospital Munich, Munich, 81377, Germany; f Bavarian Cancer Research Center (BZKF), Munich, 81377, Germany; g Radiology Department, University Hospital Nice, Nice, 06001 Nice Cedex 1, France

**Keywords:** Radiation-induced pneumonitis, *T*_2_-mapping, MR-only, Automated assessment, Radiotherapy follow-up

## Abstract

**Background and purpose:**

Radiation-induced pneumonitis (RP), a complication of lung radiotherapy, occurs at the earliest 6–12 weeks post-treatment. To assess RP, repeated computed tomography (CT)-scans post-radiotherapy are standard-of-care, but increase the patients’ dose burden and secondary cancer risk. We propose a pipeline based on magnetic resonance imaging (MRI) $T_{2}$-mapping acquired 2–3 months post-radiotherapy that provides an automated patient stratification and initial segmentation of the RP lung volume.

**Materials and methods:**

In total, 24 lung tumor patients received MRI-guided radiotherapy at a 0.35 T MR-Linac. MRI $T_{2}$-maps were retrieved from $T_{2}$-weighted images acquired at a diagnostic 1.5 T MRI-scanner 8–20 weeks post-radiotherapy. Mean baseline-corrected $T_{2}$-values were calculated in the planning target volume and the lung volume receiving≥20 Gy excluding the gross tumor volume. Their stratification potential (endpoint RP grade≥1) was assessed in a univariate receiver operating characteristic curve–area under the curve (ROC–AUC) analysis using bootstrapping. Significant differences were probed (Mann–Whitney U test, $\alpha_{\mathrm{Stats}}$=0.05). Thresholding using the maximal Youden index was utilized for the $T_{2}$-based RP segmentation. The Dice similarity coefficient (DSC), 95% Hausdorff distance (HD95), sensitivity, precision and segmentation AUC (SegAUC) were used for the comparison with the ground-truth CT-based RP segmentation.

**Results:**

RP grade≥1 was diagnosed in 15/24 patients. The $T_{2}$-values in both regions achieved significant separation of distributions (median 13.8/2.9 ms and 5.0/-2.6 ms RP/non-RP) with p-values<0.05 and AUC≥0.76. Moderate quantitative agreement was found between $T_{2}$-based and ground-truth segmentation (DSC=0.32, HD95=20.1 mm and SegAUC=0.76).

**Conclusion:**

MRI $T_{2}$-values allow an automated RP patient stratification and initial RP lung volume estimation.

## Highlights

• Investigation of MRI follow-up for assessment of radiation-induced pneumonitis (RP).
• Proposal of pipeline for automated post-radiotherapy RP patient stratification.
• Pipeline based on *T*_2_-map for automated post-radiotherapy RP patient stratification.
• First automated *T*_2_-based segmentation of lung volume affected by RP.

## Graphical abstract

**Figure:**
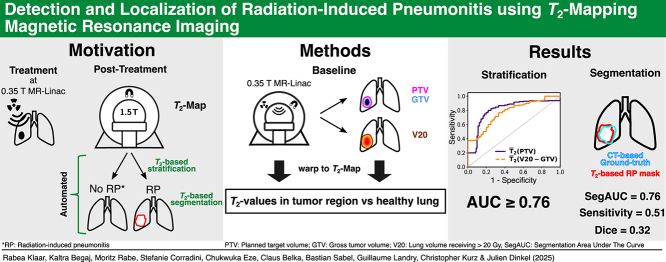

## 1 Introduction

Stereotactic body radiation therapy (SBRT) has been established as standard-of-care for inoperable non-small cell lung cancer (NSCLC) and lung metastasis [1], [2], [3]. However, due to the lung’s high radiosensitivity [4], radiation-induced pneumonitis (RP) is a considerably dose-limiting factor in thorax radiotherapy [5], [6]. It also remains a common side effect with incidences of 10%–17% for grade ≥  2 [7], [8] and up to 30%–87% when including grade 1 [9], [10] after magnetic resonance-guided radiotherapy (MRgRT). The earliest signs of (acute) RP are typically observed within 8–18 weeks after the end of radiotherapy, but can develop until about six months post-radiotherapy and potentially result in severe long-term impairment [6], [11]. Standard-of-care for assessing radiation-induced toxicities and monitoring tumor control are repeated acquisitions of follow-up computed tomography (FupCT) or PET-CT scans after the end of radiotherapy (years 1–2: every three months, years 2–4: every six months, after year 4: annual) together with the clinical presentation of the patient [9]. For patients with radiological findings, such as ground-glass opacities and consolidations [6], [12], a recent Delphi consensus study [13] suggested close monitoring with reassessment three weeks after the diagnosis or in severe cases even daily to every third day. Considering that these repeated CT- and/or PET-CT-scans require radiation dose and/or the administration of radionuclides which can amount to a considerable dose exposure for the patient [14], [15], [16], replacing these scans with radiation dose-free MRI-scans could help to decrease the non-negligible risk of radiation-induced cancers [14], [16].Recent studies have shown that $T_{2}$-weighted MR imaging is sensitive to different lung tissue patterns observable in, for example, cystic fibrosis and idiopathic pulmonary fibrosis [17], [18]. Additionally, the use of quantitative MRI and in particular $T_{2}$-mapping for the differentiation of active-inflammatory and stable-fibrotic lesions as well as the characterization of lung tissue patterns has been successfully demonstrated in non-specific interstitial pneumonia and usual interstitial pneumonia [19], [20]. The potential of $T_{2}$-mapping for the diagnosis of RP has been suggested by pre-clinical studies, as considerably increased $T_{2}$-values have been observed in RP-affected lung tissue compared to normal lung parenchyma [21], [22]. This is caused by the obliteration of air space at the air–tissue interfaces due to the infiltration of inflammatory cells inherent in RP into the intra-alveolar space, which results in a reduction of magnetic field inhomogeneities and prolonged $T_{2}$ relaxation. Considering this, investigating $T_{2}$-value changes in the high-dose region relative to healthy lung parenchyma is hypothesized to allow for the differentiation of RP and non-RP tissue.The aim of this study was to investigate the potential of $T_{2}$-mapping at a diagnostic 1.5T MRI-scanner in a clinical study for the diagnosis of RP after MRgRT at a low-field MR-Linac. For this, a pipeline for the automated stratification of patients into RP and non-RP patients is proposed along with a first, automated segmentation of the lung volume affected by RP based on the $T_{2}$-values, which could allow an initial assessment of the extent of the radiation-induced lung injury. This feasibility study could provide a first step towards an MRI-only post-radiotherapy follow-up workflow to reduce imaging-induced cancer risk, while facilitating RP diagnosis [23], [24].

## 2 Materials and methods

### 2.1 Patient cohort

Between June 2021 and December 2024, a total of 24 patients with 25 lung lesions received hypofractionated online adaptive MRgRT with a gated beam delivery at the 0.35 T MR-Linac (MRIdian, ViewRay Inc., Cleveland, Ohio) situated at the LMU University Hospital Munich and were included in this study (Table 1). Ethical approval (project number 21-0019) for this prospective study was granted by the local ethics committee and written informed consent was signed by all patients. Inclusion criteria were SBRT in at least three treatment fractions and no infracarinal lesions. One patient received simultaneous SBRT for two targets (primary tumor and metastasis).

**Table 1:** Patient characteristics for the 24 patients. Unless indicated otherwise, all numbers reported in the table are in units of patient numbers. The fractionation is given as physical dose. *GTV: Gross tumor volume, NSCLC: Non-small cell lung cancer, RP: Radiation-induced pneumonitis*.

Age [yrs]		Median		63
		Range		38-81
Sex		Male		11
		Female		13

Fractionation		3×13.5Gy		7 (29%)
		3×15.0Gy		4 (17%)
		5×8.0Gy		1 (4%)
		5×10.0Gy		1 (4%)
		8×7.5Gy		2 (8%)
		10×4.0Gy		1 (4%)
		10×5.0Gy		8 (33%)

GTV size [${\mathrm{cm}}^{3}$]		Median		9.0
		Range		1.4–71.4

Tumor location		Superior lobe left		7 (28%)
		Superior lobe right		8 (32%)
		Inferior lobe left		5 (20%)
		Inferior lobe right		5 (20%)

Type of cancer		Primary lesion		6 (24%)
		Metastasis		19 (76%)

NSCLC stage		IA-B		3
		IIA		1
		IVA-B		2

RP grade		Grade 0		9 (38%)
		Grade 1		11 (43%)
		Grade 2		4 (19%)

RP volume [${\mathrm{cm}}^{3}$]		Median		84.6
		Range		3.7–316.2

### 2.2 RP grading

As part of the standard follow-up procedure post-radiotherapy, all patients received a FupCT after a median time of eleven weeks (range: 5–25 weeks) after the end of treatment. An experienced radiologist assessed all FupCTs with respect to radiation-induced changes corresponding to RP such as consolidations and ground-glass opacities in the vicinity of the irradiated lesion. The RP grading was performed based on the National Cancer Institute Common Terminology Criteria for Adverse Events (NCI-CTCAE) version 5.0 [25] using the FupCT radiological appearance along with the patients’ general condition and potential symptoms assessed during regular follow-up. Besides the categorical RP grading, the lung volume affected by RP was segmented by the radiologist (FupCT RP mask), which served as ground-truth for the $T_{2}$-based RP segmentation task.

### 2.3 MR image acquisition and processing

**Fig. 1:**
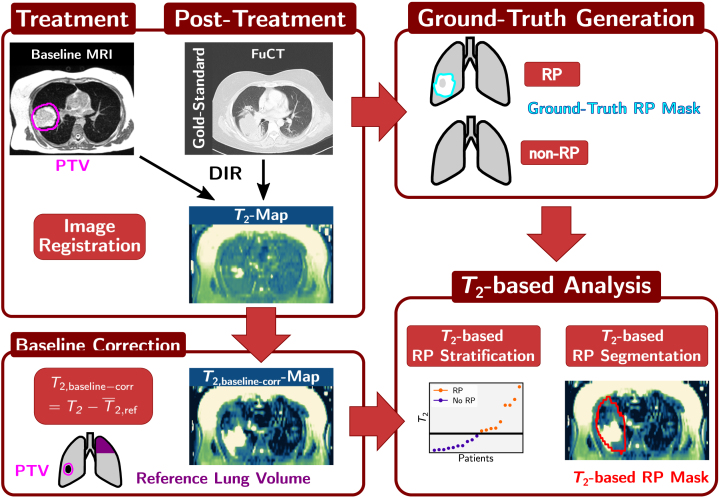
Illustration of the image acquisition and processing workflow. In addition to the baseline MRI (acquired for planning before treatment) and the standard-of-care FupCT (acquired about 2–3 months after end of treatment), $T_{2}$-weighted images at five different echo times to generate follow-up $T_{2}$-maps were obtained (top left). Based on the FupCT, RP status was assessed and the ground-truth RP masks, i.e., the segmentation of the RP extent, were delineated (top right). The baseline MRI and FupCT were deformably registered to the first $T_{2}$-weighted image and the PTV, GTV and V20 as well as the ground-truth RP mask were propagated to the $T_{2}$-map. Subtracting the mean $T_{2}$ in a healthy part of the lung with maximal distance from the tumor (${\bar{T}}_{2,\mathrm{ref}}$) from the $T_{2}$-map, baseline corrected $T_{2,\mathrm{baseline-corr}}$ were generated (bottom left). This baseline-corrected $T_{2}$-map was then used to perform $T_{2}$-based RP stratification and $T_{2}$-based RP segmentation (bottom right). *DIR: Deformable image registration, FupCT: Follow-up CT, GTV: Gross tumor volume, PTV: Planning target volume, RP: Radiation-induced pneumonitis, V20: Lung volume receiving more than 20 Gy*.

An overview of the workflow including the image acquisition and processing steps is given in Fig. 1.

#### 2.3.1 Treatment planning stage

The standard MRgRT workflow includes, besides the imaging on a fraction-by-fraction basis, a 3D baseline MRI-scan performed about one week before the start of treatment on which the target and organs-at-risk (OARs) were delineated and the dose distribution optimized. For this, a 3D MRI-scan using a balanced steady-state free precession (bSSFP) sequence was acquired for each patient in inspiration breath-hold. The planning target volume (PTV) was defined as the gross tumor volume (GTV) with an isotropic 5mm margin. The specific sequence parameters are reported in [26].

#### 2.3.2 Follow-up stage

All patients included in this study underwent an additional diagnostic follow-up MRI (FupMRI) at a 1.5T MRI-scanner (MAGNETOM Aera/SolaFit, Siemens Healthineers, Erlangen, Germany) located at the Department of Radiology of the LMU University Hospital Munich. The median time between the standard-of-care FupCT and the FupMRI was 25 days (range: 0–61 days). Utilizing an electrocardiogram-triggered single-shot fast spin-echo sequence (Table 2) with phase conjugate symmetry, $T_{2}$-weighted images of the full lung volume were acquired in diastole in an interleaved fashion with two inspiration breath-holds per volume. By repeating the acquisition with TE=[18,36,61,100,131]ms, five 3D volumes with different $T_{2}$-weighting were acquired. To generate the $T_{2}$-maps for each patient, first, the different $T_{2}$-weighted images were slice-wise, deformably registered, using open-source software ANTs [27] (antspyx package, version 0.2.9; multi-stage diffeomorphic transformation with mutual information (MI) as metric), to the TE=18ms $T_{2}$-weighted image, which served as reference for this and later registration steps. Using the registered $T_{2}$-weighted images, the logarithm of the signal for the different TEs was linearly fitted on a voxel-by-voxel basis to generate $T_{2}$-maps.

**Table 2:** The sequence parameters for the acquisition of the $T_{2}$-weighted images at the diagnostic 1.5T MRI-scanner. *FOV: Field-of-view, TE: Echo time, TR: Repetition time, SS: Single-shot, FSE: Fast spin-echo, GRAPPA: Generalized autocalibrating partial parallel acquisition*.

Acquisition dimensions		3D
Excitation mode		slice-selective
Sequence type		SS-FSE
Orientation		coronal
FOV [${\mathrm{mm}}^{2}$]		400 × 400
Nr. slices		25
Acquisition matrix size		128 × 128
Reconstructed matrix size		256 × 256
Reconstructed in-plane spatial resolution [${\mathrm{mm}}^{2}$]		1.6×1.6
Slice thickness [mm]		8.8
TE [ms]		18, 36, 61, 100, 131
TR [ms]		314
Echo spacing [ms]		3.36
Flip angle [°]		145
Receiver bandwidth [Hz/pixel]		781
Parallel imaging technique		GRAPPA
Acceleration factor		3
Nr. reference lines		32
Phase Oversampling [%]		100
Phase FOV [%]		100
Partial Fourier factor		5/8
Nr. concatenations/breath-holds		2
Total acquisition time [s]		23

#### 2.3.3 Image processing

The planned dose distribution of each patient was converted into radiobiological equivalent doses of 2Gy (EQD2) based on the linear quadratic model (α/β=3Gy) to compensate for the different fractionation schemes (Table 1) [8]. To overlay target volumes and dose distributions defined on the baseline MRI onto the follow-up $T_{2}$-maps, the baseline MRI-scans were deformably registered to the reference $T_{2}$-weighted image using open-source software Plastimatch [28] (version 1.8.0; 3D registration, multi-stage b-spline with MI). Similarly, to be able to compare the ground-truth RP segmentation with the $T_{2}$-based RP segmentation, the FupCT was deformably registered (Plastimatch, multi-stage b-spline with MI) to the reference $T_{2}$-weighted image. All target structures delineated on the baseline MRI-scan and the FupCT RP mask were propagated to the $T_{2}$-map by applying the respective deformation fields. Considering that $T_{2}$-maps were only acquired at the follow-up and not at baseline stage, a baseline-correction was performed on the $T_{2}$-map of each patient. Firstly, the lung segmentation performed on the baseline MRI was propagated to the $T_{2}$-map. Next, the propagated lung mask was divided into six sub-volumes, i.e., three per lung in cranio-caudal direction. A lung subvolume with the greatest distance from the target was selected for each patient. Including only slices in which the GTV was defined, the average $T_{2}$-value over coronal slices was calculated within this subvolume ${\bar{T}}_{2,\mathrm{ref}}^{\mathrm{GTV}}$ and used to perform the baseline-correction:

(1) $$T_{2,\mathrm{baseline-corr}}=T_{2}-{\bar{T}}_{2,\mathrm{ref}}^{\mathrm{GTV}}.$$

For $T_{2}$-based patient stratification, two parameters were defined on the baseline-corrected $T_{2}$-maps: The mean $T_{2}$-value in the PTV region (${\bar{T}}_{2,\mathrm{baseline-corr}}^{\mathrm{PTV}}$) and the mean $T_{2}$-value in the lung volume that received more than 20Gy region (V20) without the GTV region (${\bar{T}}_{2,\mathrm{baseline-corr}}^{\mathrm{V20-GTV}}$).

### 2.4 Statistical analysis and metrics

The endpoint of the stratification task was defined as the differentiation between patients with RP grade ≥  1 (RP patients) and patients with RP grade = 0 (non-RP patients) using the two $T_{2}$-based parameters. A univariate analysis employing receiver operating characteristic (ROC) curves based on systematical thresholding of the mean baseline-corrected $T_{2}$-values and the corresponding area under the curve (AUC) values were used to assess the stratification performance (RP vs non-RP) of both parameters. For a first internal validation, bootstrapping (sklearn.utils.resample package; version 1.0.1) with 5000 samples was utilized and median ROC curves were determined together with the 95% confidence intervals (CIs) and the respective AUC values. Significant differences between the RP and non-RP parameter distributions were probed with the Mann–Whitney U test ($\alpha_{\mathrm{Stats}}=0.05$). While all included patients could be used for the stratification task, five of the 15 RP patients had to be excluded from the $T_{2}$-based RP segmentation task as their FupCT and their FupMRI were acquired more than four weeks apart. As the comparability of the RP regions’ appearance in these two scans could not be guaranteed, it was decided to remove these patients from this part of the analysis. To restrict the region-of-interest for the RP segmentation, the baseline-corrected $T_{2}$-maps were masked with the V20 mask and lung mask propagated from the baseline MRI to the $T_{2}$-map. The baseline-corrected $T_{2}$-value cut-off to differentiate between voxels associated with RP and with healthy lung parenchyma was calculated from the associated maximal Youden index [29]:

(2) Youdenindex=sensitivity + specificity − 1

of the median ROC curve of the ${\bar{T}}_{2,\mathrm{baseline-corr}}^{\mathrm{PTV}}$ parameter after bootstrapping. The thresholded $T_{2}$-based RP mask was smoothed for better visualization by utilizing dilation (6 × 6 pixels kernel), followed by erosion (3 × 3 pixels kernel). To assess the performance of the $T_{2}$-based RP segmentation, the $T_{2}$-based RP mask was visually compared to the FupCT RP mask propagated to the $T_{2}$-map. For quantitative assessment, the Dice similarity coefficient (DSC), the sensitivity, the precision, the 95% Hausdorff distance (HD95) and the segmentation AUC (SegAUC) as defined in Powers et al. [30] were calculated.

## 3 Results

### 3.1 Patient stratification

**Fig. 2:**
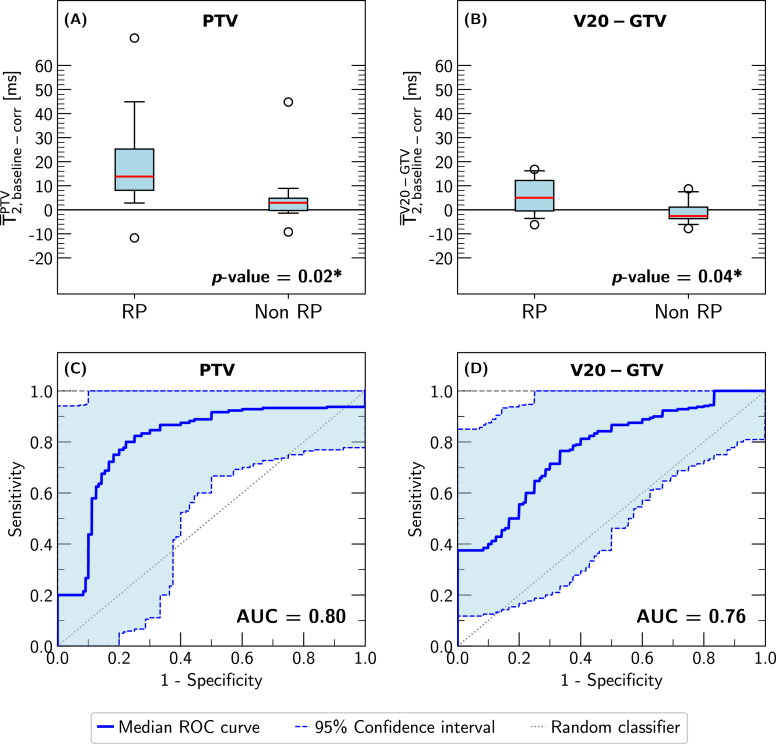
Boxplots and median ROC curves. The distributions of the RP and non-RP patients for the mean baseline-corrected $T_{2}$-values in the PTV (subplot (A)) and in the V20 without GTV region (subplot (B)) are shown. The red solid line indicates the median values, circles the outliers and the boxplot whiskers represent the 5th and 95th percentiles. Significant p-values (non-parametric Mann–Whitney U test with $\alpha_{\mathrm{Stats}}=0.05$) are denoted with an asterisk ’*’. In subplots (C) and (D), the median ROC curves (solid blue line) after 5000 bootstrapping samples are illustrated for the PTV and the V20 without GTV region. The light-blue areas bounded by the dashed blue lines denote the 95% confident intervals. The dotted line represents a random classifier.

Radiological indications for RP were found in a total of 15 patients (62%), where four patients (19%) presented with RP-related symptoms and were thus classified as RP grade 2 and eleven patients (43%) without symptoms were classified as RP grade 1. No RP indications (RP grade 0) were found for the remaining nine patients (38%).The boxplots showing the distributions of the mean baseline-corrected $T_{2}$-values in the PTV and the V20 without GTV region for the RP and non-RP patients are illustrated in Fig. 2 (subfigures (A), (B)). With p-values of 0.02 and 0.04, significant differences were found between the two patient groups for the PTV and the V20-GTV region, respectively. Looking at the distributions, increased baseline-corrected $T_{2}$-values indicated radiation-induced changes, while constant or decreased baseline-corrected $T_{2}$-values were found for non-RP patients. The results of the univariate analysis, namely the median ROC curves and the respective AUC values after bootstrapping for both investigated regions are shown in Fig. 2 (subfigures (C), (D)). Both parameters, ${\bar{T}}_{2,\mathrm{baseline-corr}}^{\mathrm{PTV}}$ and ${\bar{T}}_{2,\mathrm{baseline-corr}}^{\mathrm{V20-GTV}}$, demonstrated good stratification ability with high AUC values of 0.80 and 0.76, respectively, and thus agreement with the non-parametric Mann–Whitney U test’s findings. The maximal Youden index (Eq. (2)) of 0.69 was achieved (cut-off=7.0ms) for the mean baseline-corrected $T_{2}$-values in the PTV region and 0.61 (cut-off=0.30ms) for the V20-GTV region.

### 3.2 $T_{2}$-Based RP segmentation

**Fig. 3:**
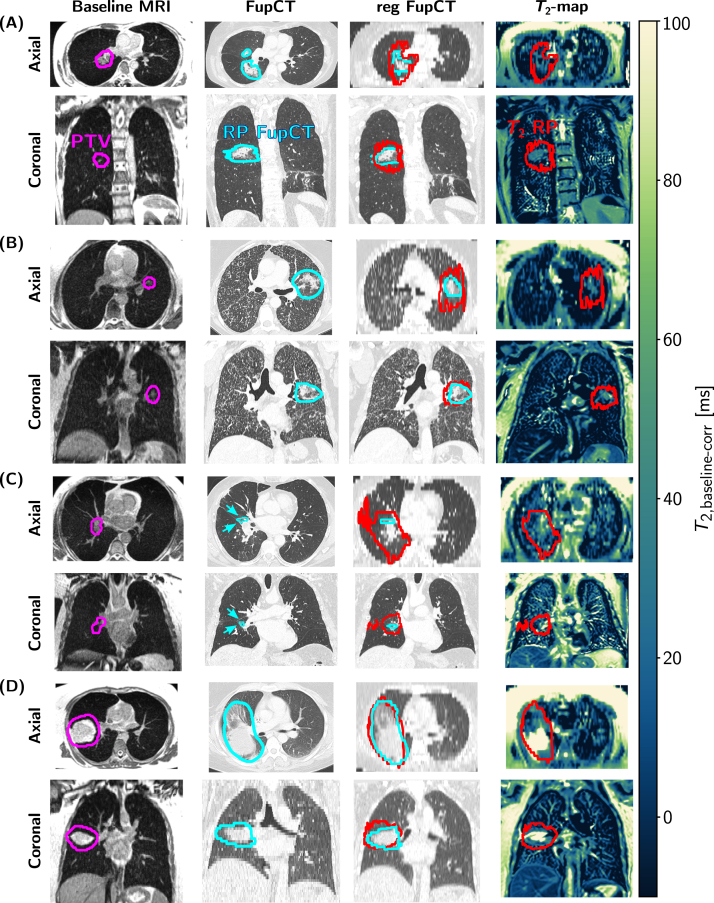
Visual segmentation results. The baseline MRI, acquired at the MR-Linac, with the corresponding PTV (pink contour), the original FupCT with ground-truth RP mask by a radiologist (light-blue contour and arrows), the baseline-corrected $T_{2}$-map with the $T_{2}$-based RP mask (red contour) and the FupCT registered to the $T_{2}$-map space (reg FupCT) with the ground-truth and $T_{2}$-based RP mask are shown for four exemplary patients (patient 1 (RP grade 2) in subfigure (A), patient 2 (RP grade 2) (B), patient 3 (RP grade 1) (C), patient 6 (RP grade 2) (D).

Four exemplary patients with varying initial tumor size and RP extent are presented in Fig. 3. In patients with average to large RP extent, a good visual agreement was found between $T_{2}$-based RP mask and FupCT-based ground-truth (subfigures (A), (B), (D)), despite a slight overestimation of the RP extent by the $T_{2}$-based RP mask for patient 2 (subfigure (B)). In patients with minor radiation-induced changes, an overestimation was observed (subfigure (C)).The quantitative results for the segmentation metrics (Section 2.4) are given in Table 3. For the DSC and the HD95, moderate agreement was found between the ground-truth and the $T_{2}$-based RP mask with median values over all patients of 0.32 and 20.1 mm, respectively. The median values over all patient of the sensitivity and precision demonstrate a limited performance with respective values of 0.51 and 0.44, respectively. A higher median SegAUC value of 0.76 was achieved. At a patient level, the best results with above average DSC, precision and SegAUC were found for patient 2 (0.59, 0.61 and 0.78) and patient 6 (0.65, 0.73 and 0.79). This agrees with the visual impression in subfigures (B) and (D) (Fig. 3).

**Table 3:** Quantitative segmentation results. The Dice similarity coefficient (DSC), sensitivity, precision, segmentation area under the curve (SegAUC) value and the 95% Hausdorff distance (HD95) were calculated for the $T_{2}$-based RP mask of the RP patients using the RP mask segmented by a radiologist on the FupCT as ground-truth.

		DSC	Sensitivity	Precision	SegAUC	HD95 [mm]
Patient 1		0.40	0.46	0.35	0.73	19.1
Patient 2		0.59	0.56	0.61	0.78	25.8
Patient 3		0.04	0.75	0.02	0.88	33.8
Patient 4		0.28	0.60	0.18	0.80	19.5
Patient 5		0.50	0.46	0.57	0.73	17.3
Patient 6		0.65	0.58	0.73	0.79	14.3
Patient 7		0.35	0.70	0.51	0.85	17.7
Patient 8		0.10	0.21	0.06	0.60	20.7
Patient 9		0.10	0.05	0.52	0.53	55.0
Patient 10		0.30	0.20	0.55	0.60	21.0

Median		0.32	0.51	0.44	0.76	20.1

## 4 Discussion

The main objective of the presented study was to explore the potential of post-radiotherapy $T_{2}$-mapping MRI to provide a method for the automated identification of RP patients and a first estimation of the RP-affected lung volume. For the stratification task, two parameters in the high-dose region (${\bar{T}}_{2,\mathrm{baseline-corr}}^{\mathrm{PTV}}$, ${\bar{T}}_{2,\mathrm{baseline-corr}}^{\mathrm{V20-GTV}}$) were defined and demonstrated promising results with median AUC values ≥  0.76 after bootstrapping. Voxel-wise thresholding using a cut-off derived from the median ROC curve’s Youden index provided a $T_{2}$-based RP mask that achieved moderate conformity with the ground-truth FupCT RP mask in terms of quantitative metrics, but overall good visual agreement especially for medium to large RP lung volumes. To the best of the authors’ knowledge, the presented study is the first to demonstrate a pipeline for the use of $T_{2}$-mapping for the assessment of radiation-induced lung injuries in lung tumor patients post-radiotherapy.Hypothesized increased $T_{2}$-values for RP patients were confirmed by significant differences between patient groups in baseline-corrected $T_{2}$-value changes in the high-dose region relative to healthy lung parenchyma. As pre-treatment $T_{2}$-maps could not be acquired and thus radiation-induced changes to other healthy lung parts or pre-existing comorbidities not assessed, a baseline-correction with mean $T_{2}$-values found in lung regions with greatest distance from the GTV was utilized. In order to limit the potential gravitational influence on $T_{2}$-values between anterior and posterior lung regions and therefore on the baseline-correction, only slices in which the GTV was defined were used. Since the ROC analysis achieved promising results and the patient cohort was small for the segmentation task, a simplified approach based on thresholding using the Youden index, which was originally developed for the assessment of a biomarker’s differentiating abilities [29], was pursued and demonstrated to be suitable for medium to large RP regions.Even though the automated patient stratification could be also performed on the $T_{2}$-weighted images (Supplementary Material 1) and the automated segmentation based on the $T_{2}$-weighted images achieved comparable median results, $T_{2}$-mapping promises, as a quantitative MRI method, a more robust approach in a multi-MRI-scanner setting. The lack of additional radiation dose and/or administration of radionuclides and thus the reduction of imaging-induced cancer risk is a clear advantage of the presented workflow over previous studies investigating SPECT/CT and PET/CT post-radiotherapy for the assessment of radiation-induced changes. In a study by Farr et al. [31], the acquisition of perfusion SPECT/CT-scans performed pre- and three months post-radiotherapy was suggested and a correlation between regional perfusion and the severity of symptoms corresponding to RP was established. Similar to our study, significant differences between RP and non-RP patients were found in specific dose regions. However, in contrast to the proposed concepts, most other studies focusing on post-radiotherapy SPECT/CT and PET/CT imaging, e.g., by Zhang et al. [32], Siva et al. [33] or Scheenstra et al. [34], provided only mere descriptions of lung perfusion changes in different lung regions over time. Apart from lacking (automated) stratification between RP and non-RP patients, none of these studies determined the extent of the radiation-affected lung volume on a voxel-by-voxel basis.Despite its novelty, the presented study is limited by the moderate quantitative performance of the $T_{2}$-based RP segmentation. Even though patients with a time difference of more than 4 weeks between FupCT and FupMRI were excluded for the segmentation task, slight differences in the RP appearance could still influence the quantitative performance of the $T_{2}$-based RP segmentation. Furthermore, considering that the $T_{2}$-based segmentations retrieved from voxel-based thresholding still show ragged boundaries after the use of dilation and erosion as smoothing operations and that quantitative metrics such as DSC and HD95 are determined by the overlap of shapes of the manually delineated ground-truth and the ragged $T_{2}$-based RP mask, an overall lower agreement is expected. These commonly used metrics to assess segmentation performance are therefore not solely indicative of the quality of the results. As the differentiation between local tumor recurrence and mass-like appearance of RP can be challenging [12], [35], these structures were included in the ground-truth RP mask if additional radiation-induced tissue changes were present on the CT-scan. These potential uncertainties in the ground-truth FupCT RP masks along with inherent uncertainties of the non-rigid image registration step to propagate these FupCT RP masks to the $T_{2}$-map and potential $T_{2}$-value deviations introduced by the linear fit of the signal’s logarithm to generate the $T_{2}$-maps [36] may further influence the qualitative and especially quantitative segmentation comparisons.However, using the pipeline proposed in this feasibility study, a first, automated stratification of RP and non-RP patients could be provided, which demonstrates a first step towards an MRI-only follow-up workflow after the end of radiotherapy. This has the potential to reduce the amount of radiation dose associated with the regular FupCT-scans especially within the first five years after radiotherapy [14], [16]. Furthermore, it could aid the sometimes challenging diagnosis/scoring of RP [23], [24] and provide, together with an MRI-based RP prediction [26], a closer MRI-only RP patient and disease monitoring. In combination with the first estimation of the RP extent found with the $T_{2}$-based RP segmentation, this could trigger medical interventions based on RP severity. In further investigations, characterizing and differentiating between different tissue types, i.e., RP, radiation-induced fibrosis, remaining and/or recurring tumor and healthy tissue, based on their respective $T_{2}$-values, as suggested by Shioya et al. [21], combined with complementary (functional) imaging methods [26] could be a promising approach in a larger, multi-center study with wider variety in post-radiotherapy tissue changes to improve the MRI-based RP segmentation and to reduce the need for follow-up (PET-)CT-scans. The performance comparison of $T_{2}$-mapping with other suggested and potentially RP- or rather inflammation-sensitive MRI-based methods such as diffusion-weighted imaging [37] and $T_{1}$-mapping [38] or CT-like ultrashort TE sequences [39] were outside the scope of this work, but are worth considering. In this study, a pipeline was proposed to stratify patients into RP and non-RP patient groups and to provide a first localization and estimated extent of the RP-affected lung volume based on 3D $T_{2}$-maps 2–3 months after the end of lung MRgRT. Good stratification capabilities were found for mean baseline-corrected $T_{2}$-values in the high-dose region (PTV and V20 without GTV) with significant p-values ≤  0.04 and AUC values ≥  0.76 achieved after bootstrapping in a univariate analysis. Moderate quantitative, but promising visual agreement of the $T_{2}$-based segmentation with the ground-truth FupCT RP mask was found.

## CRediT authorship contribution statement

**Rabea Klaar:** Data curation, Investigation, Software, Formal analysis, Visualization, Conceptualization, Writing – original draft. **Kaltra Begaj:** Data curation, Writing – review & editing. **Moritz Rabe:** Data curation, Investigation, Conceptualization, Writing – review & editing. **Stefanie Corradini:** Project administration, Supervision, Writing – review & editing. **Chukwuka Eze:** Project administration, Supervision, Writing – review & editing. **Claus Belka:** Project administration, Resources. **Bastian Sabel:** Resources, Funding acquisition. **Guillaume Landry:** Conceptualization, Investigation, Methodology, Writing – review & editing. **Christopher Kurz:** Conceptualization, Methodology, Investigation, Writing – review & editing. **Julien Dinkel:** Conceptualization, Methodology, Investigation, Project administration, Funding acquisition.

## Declaration of competing interest

The authors declare the following financial interests/personal relationships which may be considered as potential competing interests: The Department of Radiation Oncology of the LMU University Hospital Munich has a research agreement with Brainlab and Elekta. The Department of Radiology of the LMU University Hospital Munich has a master research agreement with Siemens Healthineeers.

## Appendix A Supplementary data

MMC S1 Mean signal intensities in the PTV and V20-GTV region of the $T_{2}$-weighted images acquired at five different echo times.

## References

[1] Mayinger M., Kotecha R., Sahgal A., Kim M.S., Lo S.S., Louie A.V. (2023). Stereotactic body radiotherapy for lung oligo-metastases: Systematic review and international stereotactic radiosurgery society practice guidelines. Lung Cancer.

[2] Yan M., Louie A.V., Kotecha R., Ashfaq Ahmed M., Zhang Z., Guckenberger M. (2023). Stereotactic body radiotherapy for ultra-central lung tumors: A systematic review and meta-analysis and international stereotactic radiosurgery society practice guidelines. Lung Cancer.

[3] Postmus P., Kerr K., Oudkerk M., Senan S., Waller D., Vansteenkiste J. (2017). Early and locally advanced non-small-cell lung cancer (NSCLC): ESMO clinical practice guidelines for diagnosis, treatment and follow-up. Ann Oncol.

[4] Giuranno L., Ient J., De Ruysscher D., Vooijs M.A. (2019). Radiation-induced lung injury (RILI). Front Oncol.

[5] Hanania A.N., Mainwaring W., Ghebre Y.T., Hanania N.A., Ludwig M. (2019). Radiation-induced lung injury: Assessment and management. Chest.

[6] Käsmann L., Dietrich A., Staab-Weijnitz C.A., Manapov F., Behr J., Rimner A. (2020). Radiation-induced lung toxicity–cellular and molecular mechanisms of pathogenesis, management, and literature review. Radiat Oncol.

[7] Kang H., Kwak Y., Kim M., Lee S. (2022). Application of real-time MRI-guided linear accelerator in stereotactic ablative body radiotherapy for non-small cell lung cancer: one step forward to precise targeting. J Cancer Res Clin Oncol.

[8] Finazzi T., Haasbeek J.A., Spoelstra F.O.B., Palacios M.A., Admiraal M.A., Bruynzeel A.M.E. (2020). Clinical outcomes of stereotactic MR-guided adaptive radiation therapy for high-risk lung tumors. Int J Radiat Oncol Biol Phys.

[9] Hering S., Nieto A., Marschner S., Hofmaier J., Schmidt-Hegemann N.S., da Silva Mendes V. (2024). The role of online MR-guided multi-fraction stereotactic ablative radiotherapy in lung tumours. Clin Transl Radiat Oncol.

[10] Qiao Q., Zhu W., Tian C., Shi X., Xie P., Li Z. (2025). Real-time magnetic resonance imaging guided accelerator in stereotactic body radiation therapy for non-small cell lung cancer. J Thorac Dis.

[11] Arroyo-Hernández M., Maldonado F., Lozano-Ruiz F., Muñoz-Montaño W., Nuñez-Baez M., Arrieta O. (2021). Radiation-induced lung injury: current evidence. BMC Pulm Med.

[12] Ronden M., Palma D., Slotman B., Senan S. (2018). Advanced radiation technology committee of the international association for the study of lung cancer. Brief report on radiological changes following stereotactic ablative radiotherapy (SABR) for early-stage lung tumors: A pictorial essay. J Thorac Oncol.

[13] Voruganti Maddali I.S., Cunningham C., McLeod L., Bahig H., Chaudhuri N., Chua, K. L.M. (2024). Optimal management of radiation pneumonitis: Findings of an international delphi consensus study. Lung Cancer.

[14] Smith-Bindman R., Chu P.W., Azman Firdaus H., Stewart C., Malekhedayat M., Alber S. (2025). Projected lifetime cancer risks from current computed tomography imaging. JAMA Intern Med.

[15] Rampinelli C., De Marco P., Origgi D., Maisonneuve P., Casiraghi M., Veronesi G. (2017). Exposure to low dose computed tomography for lung cancer screening and risk of cancer: secondary analysis of trial data and risk-benefit analysis. BMJ.

[16] Cao C.F., Ma K.L., Shan H., Liu T.F., Zhao S.Q., Wan Y. (2022). Ct scans and cancer risks: A systematic review and dose–response meta-analysis. BMC Cancer.

[17] Benlala I., Hocke F., Macey J., Bui S., Berger P., Laurent F. (2020). Quantification of MRI T2-weighted high signal volume in cystic fibrosis: A pilot study. Radiology.

[18] Benlala I., Albat A., Blanchard E., Macey J., Raherison C., Benkert T. (2021). Quantification of MRI T2 interstitial lung disease signal-intensity volume in idiopathic pulmonary fibrosis: A pilot study. J Magn Reson Imaging.

[19] Buzan M.T., Eichinger M., Kreuter M., Kauczor H.U., Herth F., Warth A. (2015). T2 mapping of CT remodelling patterns in interstitial lung disease. Eur Radiol.

[20] Buzan M.T.A., Wetscherek A., Heussel C.P., Kreuter M., Herth F.J., Warth A. (2017). Texture analysis using proton density and T2 relaxation in patients with histological usual interstitial pneumonia (UIP) or nonspecific interstitial pneumonia (NSIP). PLoS One.

[21] Shioya S., Haida M., Ono Y., Fukuzaki M., Matsu-ura Y., Tsuda M. (1994). Tissue characterization of pneumonia and irradiated rat lungs with magnetic resonance relaxation times. Magn Reson Imaging.

[22] Shioya S., Tsuji C., Kurita D., Katoh H., Tsuda M., Haida M. (1997). Early damage to lung tissue after irradiation detected by the magnetic resonance T2 relaxation time. Radiat Res.

[23] Kocak Z., Evans E.S., Zhou S.M., Miller K.L., Folz R.J., Shafman T.D. (2005). Challenges in defining radiation pneumonitis in patients with lung cancer. Int J Radiat Oncol Biol Phys.

[24] Yirmibesoglu E., Higginson D.S., Fayda M., Rivera M.P., Halle J., Rosenman J. (2012). Challenges scoring radiation pneumonitis in patients irradiated for lung cancer. Lung Cancer.

[25] NCI (2017). https://ctep.cancer.gov/protocolDevelopment/electronic_applications/ctc.htm.

[26] Klaar R., Rabe M., Stüber A.T., Hering S., Corradini S., Eze C. (2024). MRI-based ventilation and perfusion imaging to predict radiation-induced pneumonitis in lung tumor patients at a 0.35t MR-linac. Radiother Oncol.

[27] Avants B.B., Tustison N.J., Song G., Cook P.A., Klein A., Gee J.C. (2011). A reproducible evaluation of ANTs similarity metric performance in brain image registration. NeuroImage.

[28] Sharp GC, Li R, Wolfgang J, Chen G, Peroni M, Spadea MF, et al. Plastimatch: an open source software suite for radiotherapy image processing. In: Proceedings of the XVI’th International Conference on the use of Computers in Radiotherapy. 2010.

[29] Ruopp M.D., Perkins N.J., Whitcomb B.W., Schisterman E.F. (2008). Youden index and optimal cut-point estimated from observations affected by a lower limit of detection. Biom J.

[30] Powers D. (2011). Evaluation: from precision, recall and F-measure to ROC, informedness, markedness and correlation. J Mach Learn Technol.

[31] Farr K.P., Møller D.S., Khalil A.A., Kramer S., Morsing A., Grau C. (2015). Loss of lung function after chemo-radiotherapy for NSCLC measured by perfusion SPECT/CT: correlation with radiation dose and clinical morbidity. Acta Oncol.

[32] Zhang J., Ma J., Zhou S., Hubbs J.L., Wong T.Z., Folz R.J. (2010). Radiation-induced reductions in regional lung perfusion: 0.1–12 year data from a prospective clinical study. Int J Radiat Oncol Biol Phys.

[33] Siva S., Hardcastle N., Kron T., Bressel M., Callahan J., MacManus M.P. (2015). Ventilation/perfusion positron emission tomography—Based assessment of radiation injury to lung. Int J Radiat Oncol Biol Phys.

[34] Scheenstra A.E., Rossi M.M., Belderbos J.S., Damen E.M., Lebesque J.V., Sonke J.J. (2013). Local dose–effect relations for lung perfusion post stereotactic body radiotherapy. Radiother Oncol.

[35] Ruysscher D.D., Wauters E., Jendrossek V., Filippi A.R., Revel M.P., Faivre-Finn C. (2025). Diagnosis and treatment of radiation induced pneumonitis in patients with lung cancer: An ESTRO clinical practice guideline. Radiother Oncol.

[36] Otto R., Ferguson M.R., Marro K., Grinstead J.W., Friedman S.D. (2011). Limitations of using logarithmic transformation and linear fitting to estimate relaxation rates in iron-loaded liver. Pediatr Radiol.

[37] Milito C., Pulvirenti F., Serra G., Valente M., Pesce A.M., Granata G. (2015). Lung magnetic resonance imaging with diffusion weighted imaging provides regional structural as well as functional information without radiation exposure in primary antibody deficiencies. J Clin Immunol.

[38] Yang S., Shan F., Yan Q., Shen J., Ye P., Zhang Z. (2020). A pilot study of native t1-mapping for focal pulmonary lesions in 3.0 t magnetic resonance imaging: size estimation and differential diagnosis. J Thorac Dis.

[39] Dournes G., Yazbek J., Benhassen W., Benlala I., Blanchard E., Truchetet M.E. (2018). 3D ultrashort echo time MRI of the lung using stack-of-spirals and spherical k-space coverages: Evaluation in healthy volunteers and parenchymal diseases. J Magn Reson Imaging.

