# Supplementary material for: Detection and localization of radiation-induced pneumonitis using T2-mapping magnetic resonance imaging
Source: Phys Imaging Radiat Oncol. 2025 Nov 29;36:100878. doi: 10.1016/j.phro.2025.100878 (PMC12720034; doi:10.1016/j.phro.2025.100878)
Supplement: MMC S1 — Mean signal intensities in the PTV and V20-GTV region of the T2-weighted images acquired at five different echo times. [file mmc1.pdf]

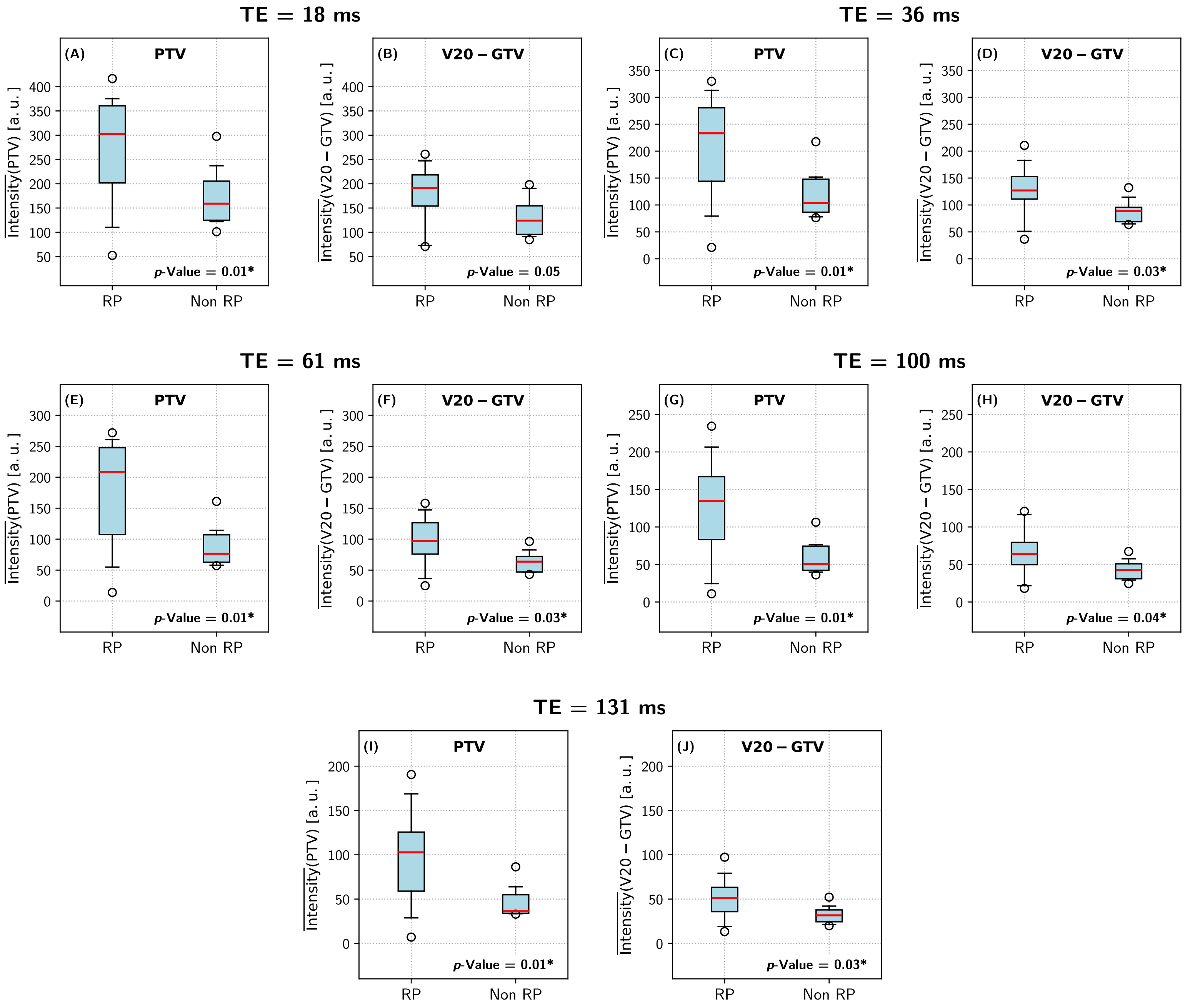

Supplementary Figure 1: The distributions of the radiation-induced pneumonitis (RP) and non-RP patients for mean signal intensities in the planning target volume (PTV) (Subfigures (A),(C),(E),(G),(I)) and the lung volume receiving ≥ 20 Gy (V20) without the gross tumor volume (GTV) (Subfigures (B),(D),(F),(H),(J)) are shown for T2-weighted images acquired with five different echo times (TE=[18, 36, 61, 100, 131] ms). Significant p-values (non-parametric Mann-Whitney U test with α_{Stats}=0.05) are denoted with an asterisk ‘*’. Median values are indicated by the red solid lines and outliers by circles. The boxplot whiskers represent the 5th and 95th percentiles.
